# *Scaphesthes zhejiangensis*, a New Species of Shoveljaw Carp (Teleostei, Cypriniformes) from Zhejiang Province, Southeast China †

**DOI:** 10.3390/ani16081176

**Published:** 2026-04-12

**Authors:** Ya-Xin Zheng, Qing-Ping Lian, Ming-Wei Zhou, Jia-Jun Zhou, Jin-Quan Yang, Ju-Lin Yuan

**Affiliations:** 1 Shanghai Universities Key Laboratory of Marine Animal Taxonomy and Evolution, Shanghai Ocean University, Shanghai 201306, China; 17763557351@139.com (Y.-X.Z.); 13402115117@163.com (M.-W.Z.); 2 Agriculture Ministry Key Laboratory of Healthy Freshwater Aquaculture, Key Laboratory of Fresh Health and Nutrition of Zhejiang Province, Zhejiang Institute of Freshwater Fisheries (Zhejiang Freshwater Fishery Environmental Monitoring Station), Huzhou 313001, China; lianqp2001@163.com; 3 Zhejiang Forest Resource Monitoring Center, Hangzhou 310020, China; cnwaters@foxmail.com; 4 Zhejiang Forestry Survey Planning and Design Company Limited, Hangzhou 310020, China

**Keywords:** Acrossocheilinae, cytochrome *b*, molecular phylogeny, *Onychostoma*, taxonomy

## Abstract

A new species of shoveljaw carp, *Scaphesthes zhejiangensis*, is described from the Qiantang River basin and three independent rivers in Zhejiang Province, Southeast China. It is distinguished from other *Scaphesthes* species by the following combination of characteristics: a long, slender body (depth 19.9–22.2% SL); 46–49 lateral-line scales; 15–17 pre-dorsal scales; a short head (depth 66.8–73.3% HL); a wide mouth (width 36.2–45.3% HL); elongated maxillary barbels shorter than one-third of the eye diameter; reduced but visible rostral barbels; a slender, smooth last simple dorsal ray; and the absence of a longitudinal black stripe along the lateral line. Molecular phylogenetic analysis based on the mitochondrial cytochrome *b* (cyt *b*) gene supported the monophyly of *S. zhejiangensis* sp. nov., which formed a clade with *S. virgulatum*, *S. macrolepis*, and the *S. barbatum* species complex.

## Simple Summary

We describe a new species of shoveljaw carp, *Scaphesthes zhejiangensis*, discovered in several river basins of Zhejiang Province, southeastern China. Previously mistaken for the morphologically similar *S. barbatulum*, it is now recognized as a distinct species. This finding underscores the rich yet still largely unexplored fish diversity in eastern China’s freshwater ecosystems, contributing to our understanding of the region’s biogeography and aiding in the conservation of this specialized fish group.

## 1. Introduction

In the revised classification of Cypriniformes, the subfamily Acrossocheilinae has been established as a distinct taxonomic unit within Cyprinidae, comprising the genera *Folifer* Wu, 1977, *Acrossocheilus* Oshima, 1919, and *Onychostoma* Günther, 1896 [1]. This subfamily, representing an independent diploid lineage, encompasses approximately 50 species of small to medium-sized benthic cyprinid fishes that primarily inhabit hill streams in eastern and southern China as well as the Indochinese Peninsula [2,3,4]. Morphologically, these genera are differentiated by lip structure: *Folifer* has thick, fleshy lips with a prominent median lobe; *Acrossocheilus* exhibits thin to moderately thick lips; and *Onychostoma* is characterized by a horny sheath on the lower jaw, earning it the common name “shoveljaw carp” [4].

The taxonomic status of *Onychostoma* remains contentious in traditional morphological studies [5,6,7]. The framework recognizing *Onychostoma* as an independent genus [7] was once widely accepted and has been followed in much of the subsequent literature [8,9]. However, molecular studies have shown that *Onychostoma* sensu lato is non-monophyletic [3,4,10,11,12,13,14]. Integrative morphological and phylogenetic analyses using three mitochondrial and one nuclear gene marker reclassified the group into four genera (*Onychostoma* sensu stricto, *Rhomboidalbarbus*, *Leptcaudalbarbus*, and *Scaphesthes*) [13]. Our study adopts this classification [13], assigning eight species to *Scaphesthes*.

Zhejiang Province, with its humid subtropical climate and mountainous terrain, provides suitable habitats for hillstream fishes. Specimens of shoveljaw carps from this region were initially identified as *Onychostoma barbatulum* [8], later reclassified as *Scaphesthes barbatulum* [4,13]. During examinations of specimens from the Qiantang River basin and three independent river systems in eastern Zhejiang, we encountered individuals morphologically distinct from *S. barbatulum*. Integrative morphological and molecular analyses confirmed these specimens represent a previously unrecognized species, formally described herein.

## 2. Materials and Methods

### 2.1. Sample Collection and Morphological Analysis

**Figure 1:**
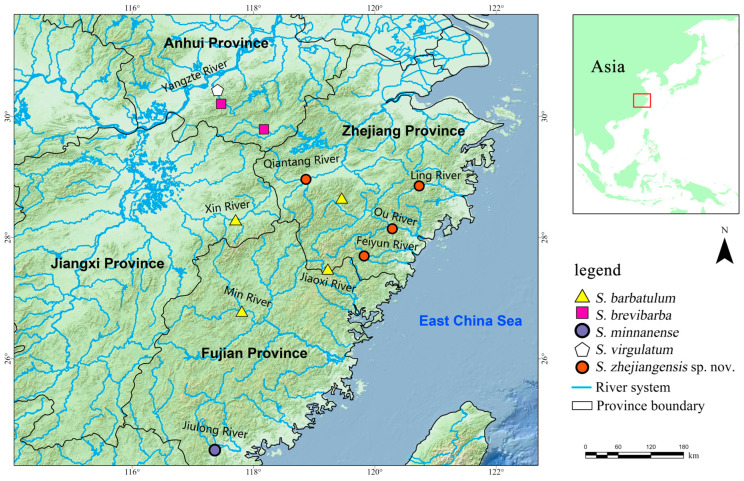
Map illustrating the sampling localities of *Scaphesthes zhejiangensis* sp. nov. and its four sympatric or allopatric congeners examined in this study.

Specimens were collected from multiple locations in Zhejiang Province, China, including Quzhou, Taizhou, Lishui, and Wenzhou (Figure 1; see Table S1 for detailed sampling information). The right pectoral fin of each specimen was preserved in 95% ethanol for molecular analysis. The remainder of each specimen was fixed in 10% formaldehyde solution for three days and then transferred to 75% ethanol for long-term storage and morphological examination. All voucher specimens are deposited at Shanghai Ocean University, Shanghai, China (SHOU). Comparative material examined included specimens of *Scaphesthes barbatulum*, *S. brevibarba*, *S. minnanense*, *S. virgulatum*, the *S. barbatum* species complex, *S. lini*, and *S. macrolepis* (Table 1 and Table 2).

Morphometric measurements, meristic counts, and descriptive protocols followed Armbruster [15]. All linear measurements were taken point-to-point using digital calipers (precision: 0.1 mm) and are presented as percentages of standard length (SL) or head length (HL). Eye diameter was measured as the greatest horizontal diameter of the exposed eyeball (note that this differs from the “orbit length” sensu Armbruster [15]). Cephalic features were examined under a stereomicroscope.

### 2.2. DNA Extraction, PCR Amplification and Phylogenetic Analysis

Genomic DNA was extracted from fin clips using an Ezup Column Animal Genomic DNA Purification Kit (Sangon Biotech Co., Ltd., Shanghai, China). The mitochondrial cytochrome *b* (cyt *b*) gene was amplified via polymerase chain reaction (PCR) using primers L14724 and H15915 [16]. The PCR protocol consisted of an initial denaturation at 94 °C for 4 min; followed by 30 cycles of denaturation at 94 °C for 30 s, annealing at 54 °C for 45 s, and extension at 72 °C for 1 min; and a final extension at 72 °C for 5 min. PCR products were verified by 1.5% agarose gel electrophoresis and sent for bidirectional Sanger sequencing at Sangon Biotech Co., Ltd. (Shanghai, China).

Sequences were assembled and edited using SeqMan software (DNASTAR, v7.1.0) [17]. Additional cyt *b* sequences for congeneric and outgroup species were retrieved from the NCBI database (Table 1). Phylogenetic analyses were conducted using PhyloSuite (v 2.0) [18]. Sequence alignment was performed with MAFFT (v7.520) under the automatic strategy and normal alignment mode [19]. The best-fit nucleotide substitution models for Maximum Likelihood (ML) and Bayesian Inference (BI) analyses were selected using ModelFinder (v 2.2.0) based on the Bayesian Information Criterion (BIC) [20]. The ML tree was constructed with IQ-TREE (v2.2.0) using the TIM2+F+G4 model, with all other parameters set to default [21]. The BI tree was constructed using MrBayes (v.3.2.6) with the GTR+F+G4 model, running two parallel Markov Chain Monte Carlo (MCMC) analyses for 1,000,000 generations, sampling every 100 generations [22]. The MCMC analysis was considered to have converged, as the average standard deviation of split frequencies dropped below 0.01 (final value = 0.005519). The first 25% of trees were discarded as burn-in. Resulting phylogenetic trees were visualized and annotated using TVBOT (v 2.6.1) [23]. Genetic distances were calculated using MEGA (v.10.0) with the Kimura 2-parameter model, computing average interspecific genetic distances [24].

## 3. Results

### 3.1. Taxonomic Account

***Scaphesthes zhejiangensis* Zheng, Zhou & Yang, sp. nov.**

**Figure 2:**
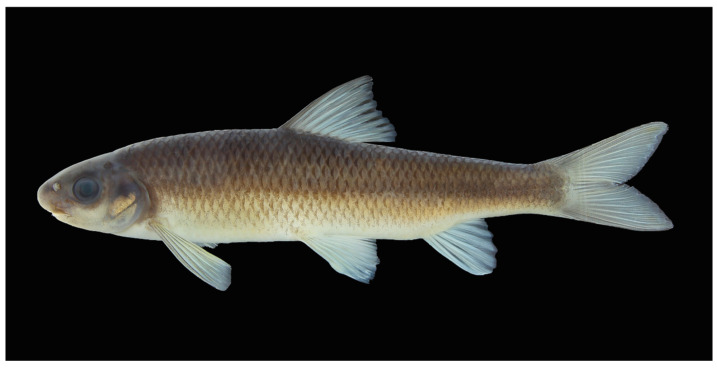
*Scaphesthes zhejiangensis* sp. nov., SHOU202505001, holotype, male, 141.5 mm SL.

**Figure 3:**
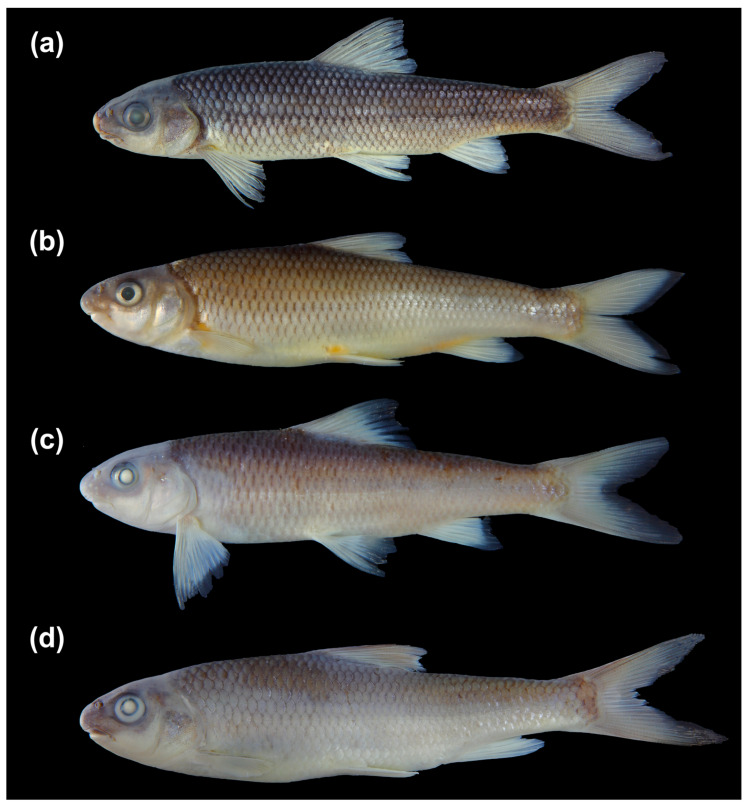
Morphological comparison of four *Scaphesthes* species: (**a**) *S. zhejiangensis* sp. nov., (**b**) *S. brevibarba*, (**c**) *S. minnanense*, and (**d**) *S. barbatulum*.

**Figure 5:**
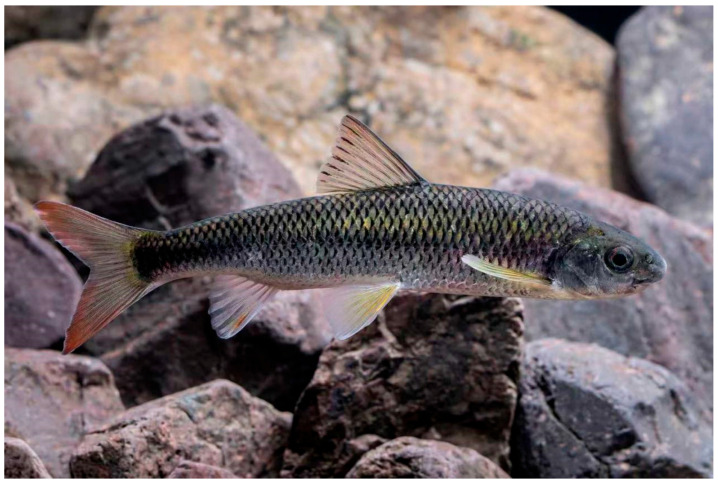
*Scaphesthes zhejiangensis* sp. nov., a live individual from Qingtian County, Lishui City, Zhejiang Province, China.

Figure 2, Figure 3a and Figure 5.

**Note on authorship.** The authority “Zhou” in the binomen refers to **Jia-Jun Zhou**.

**Holotype.** SHOU202505001, male, adult, 141.5 mm SL (Figure 2), collected from Qingtian County, Lishui City, Zhejiang Province (Oujiang River, 28.1355 °N, 120.3058 °E) in May 2025, by Jia-Jun Zhou and Ye Chen.

**Paratypes.** SHOU202403001–SHOU202403004, SHOU202502001, SHOU202505002, 6 specimens, 54.9–113.1 SL, collected by Jia-Jun Zhou & Ye Chen in March 2024, February 2025 and May 2025 from the same locality as the holotype; SHOU202408001–SHOU202408004, 4 specimens, 70.3~78.6 mm SL, collected by Jia-Jun Zhou in August 2024, in Hunan Town, Qujiang District, Quzhou City, Zhejiang Province (Qiantang River) (28.7092 °N, 118.8362 °E); SHOU202107001–SHOU202107005, 5 specimens, 83.4~86.9 mm SL, collected by Jia-Jun Zhou & Wei Sun in July 2021, in Taishun County, Wenzhou City, Zhejiang Province (Feiyun River) (27.7143 °N, 119.6577 °E); SHOU202106001–SHOU202106003, 3 specimens, 103.5~113.1 mm SL, collected by Jia-Jun Zhou & Wei Sun in June 2021, in Xianju County, Taizhou City, Zhejiang Province (Lingjiang River) (28.6676 °N, 120.5716 °E).

**Figure 4:**
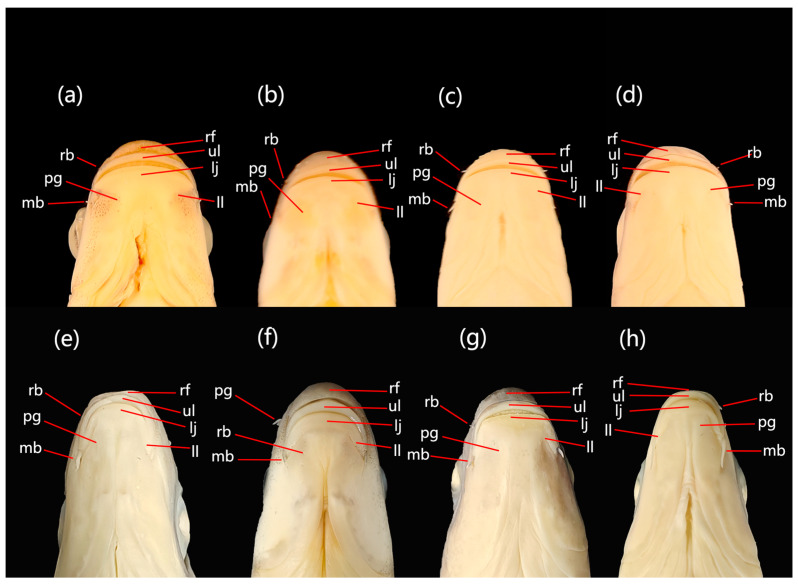
Comparison of the cephalic region (ventral part) among *Scaphesthes zhejiangensis* sp. nov. (**a**), *S. brevibarba* (**b**), *S. minnanense* (**c**), *S. barbatulum* (**d**), *S. barbatum* (**e**), *S. virgulatum* (**f**), *S. macrolepis* (**g**) and *S. lini* (**h**). Abbreviations: rf, rostral fold; rb, rostral barbels; ul, upper lip; pg, postlabial groove; lj, lower jaw; mb, maxillary barbels; ll, lower lip.

**Diagnosis.** *Scaphesthes zhejiangensis* sp. nov. is distinguished from its sympatric or parapatric congeners (*S. barbatulum*, *S. brevibarba*, *S. minnanense*) by the following combination of characters (Table 1, Figure 3 and Figure 4a–d): body longer and more slender (depth 19.9–22.2% SL vs. >22.7%); 46–49 lateral-line scales (vs. 44–45 in *S. minnanense*); 15–17 pre-dorsal scales (vs. ≤15 in *S. barbatulum* and *S. minnanense*); maxillary barbels elongated but shorter than 1/3 of eye diameter (vs. papilliform in *S. brevibarba*; longer than 1/3 in *S. minnanense*); rostral barbels papilliform (vs. minuscule in *S. brevibarba*; well-developed in *S. minnanense*).

The new species is further distinguished from allopatric congeners (*S. barbatum* complex, *S. lini*, *S. virgulatum*, *S. macrolepis*) by the following (Table 2, Figure 4e–h): a shorter head (depth 66.8–73.3% HL vs. <64%); a wider mouth (width 36.2–45.3% HL vs. <36.0%); maxillary barbels shorter than 1/3 of eye diameter (vs. ≥1/2 eye diameter in *S. barbatum*, *S. lini*, and *S. virgulatum*); a slender, smooth last simple dorsal ray (vs. strong and serrated in *S. lini* and *S. virgulatum*); absence of a longitudinal black stripe along the lateral line (vs. present in *S. barbatum*, *S. lini*, and *S. virgulatum*); and 46–49 lateral-line scales (vs. 50–53 in *S. macrolepis*).

**Description.** Morphometric and meristic data for 19 specimens are provided in Table 3. Body elongated, laterally compressed, and narrow, with the greatest depth anterior to the dorsal-fin origin. Head short, conical, and slightly compressed; head length less than body depth. Eyes moderately large, positioned dorsolaterally. Snout blunt. Mouth inferior, its opening slightly arched; a horny sheath present on the lower jaw. Maxillary barbels elongated, shorter than 1/3 of eye diameter. Rostral barbels reduced, papilliform.

Dorsal-fin rays iii,8; the last simple dorsal ray slender and smooth. Pectoral-fin rays i,15–16, their tips extending to a point anterior to the dorsal-fin origin. Pelvic-fin rays i,8–9, their origin posterior to the dorsal-fin origin. Anal-fin short, rays iii,5. Caudal fin forked, lobes pointed and nearly symmetrical.

Body covered with cycloid scales, including the pectoral and abdominal regions. Lateral line complete, descending to above the pectoral-fin tip before continuing horizontally to the caudal peduncle. Lateral-line scales 46–49; scale rows: 6–7 above lateral line, 3–4 below; pre-dorsal scales 15–17; circumpeduncular scales 16.

Live coloration (Figure 5): head and body greyish-green, abdomen silvery-white. The exposed base of each scale on the dorsum and flanks is black. Median fins tinged with red, bearing dark stripes parallel to the fin rays. Paired fins yellowish.

Preserved coloration (fixed in formalin and stored in alcohol) (Figure 2): Body dark grey, abdomen pale yellow. All fins (dorsal, caudal, anal, pectoral, and pelvic) pale grey to whitish, with pigmentation concentrated along the fin rays, giving a translucent appearance to the interradial membranes. Each scale exhibits dark grey-black edges and pale grey to whitish centers.

**Distribution.** Currently known from the Qiantang River basin and three independent coastal river systems (Lingjiang, Feiyun, and Oujiang Rivers) draining into the East China Sea in Zhejiang Province, Southeast China (Figure 1).

**Habitat.** The new species typically inhabits streams with transparent water, a moderate flow regime, and a substrate composed predominantly of gravel (Figure 5).

**Etymology.** The specific epithet zhejiangensis is the Latinized form of the Chinese Pinyin “Zhejiang,” referring to Zhejiang Province, where the newly described species is currently known to occur. For consistency, we propose the corresponding Chinese common name 浙江铲颌鱼 (Zhèjiāng Chǎnhéyú).

### 3.2. Phylogenetic Analysis

The phylogenetic analysis included 61 cyt *b* sequences, with *Spinibarbus hollandi* as the outgroup. The dataset comprised 18 new sequences of *S. zhejiangensis*, 11 from four congeneric species, and 31 sequences from GenBank representing all genera of Acrossocheilinae (except *Hongiastoma*) (Table S1). The aligned sequence length was 1141 bp.

**Figure 6:**
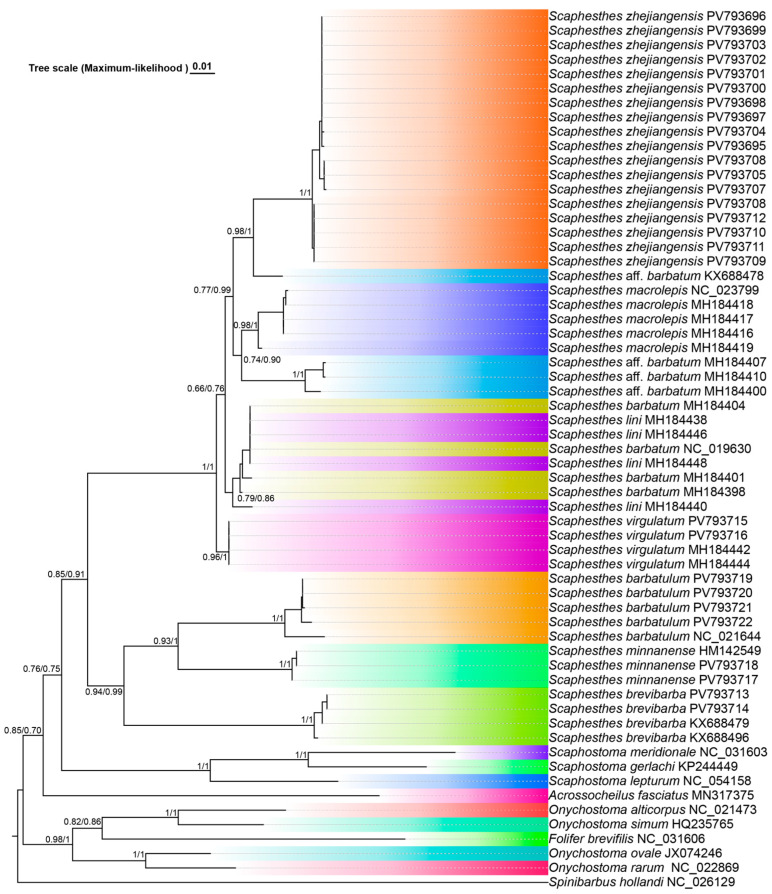
Phylogenetic tree of *Scaphesthes* and related genera inferred from the mitochondrial cyt *b* gene. The topology shown was constructed using maximum likelihood (ML); Bayesian inference (BI) recovered an identical topological structure. Nodal support values are indicated as ML bootstrap proportions and BI posterior probabilities (ML/BI). Intraspecific node supports are not shown. Branch lengths (tree scale) are estimated under the ML framework. Different colors represent different species.

As shown in Figure 6, the topologies inferred from maximum likelihood (ML) and Bayesian inference (BI) were congruent, with all species-level nodes receiving moderate to strong support (ML ≥ 0.79, BI ≥ 0.86). All samples of the new species formed a monophyletic clade, which was recovered as the sister group to *S.* aff. *barbatum* KX688478. The sister clade to this lineage comprised *S. macrolepis* and *S.* aff. *barbatum* (MH184407, MH184410, MH184400). Multiple sequences of *S. lini* and *S. barbatum* were identical and clustered together, suggesting that they should be considered as the same species from a phylogenetic perspective. This clade formed a sister group to the aforementioned two clades, albeit with relatively low nodal support (ML: 0.66, BI: 0.76). All of the above clades together constituted the sister group of *S. virgulatum*. All species of *Scaphesthes* formed a monophyletic group with moderate support (ML: 0.76, BI: 0.75). The sister group of *Scaphesthes* was identified as *Acrossocheilus*. The genus *Folifer* was recovered nested within *Onychostoma*, rendering *Onychostoma* non-monophyletic.

The intraspecific genetic distance within *S. zhejiangensis* sp. nov. was less than 0.6%. The new species showed interspecific distances of 3.9–4.9% to species within its own clade, and 11.7–14.0% to species in the other clade. Notably, the genetic divergence between *S. zhejiangensis* sp. nov. and the morphologically similar *S. barbatulum* was 11.7% (Table S2).

## 4. Discussion

Shoveljaw carps, historically classified under the broad concept *Onychostoma* sensu lato, are widely distributed in hillstream habitats across East and Southeast Asia. Molecular phylogenetic studies consistently reject the monophyly of this broadly defined group [3,4,10,11,13], prompting proposals to subdivide it. Song [13] and Hoang et al. [4] proposed alternative four-genus classification systems. In this study, we adopt Song’s [13] taxonomic framework for the genus *Scaphesthes*, which encompasses eight species, rather than the more restrictive definition proposed by Hoang et al. [4], who assigned *S. barbatum* and related species to a separate genus, *Angustistoma*. Our decision is based on morphological observations indicating that the diagnostic characters proposed by Hoang et al. [4]—such as mouth width, snout shape, and lateral scale counts—are not consistently diagnostic across all relevant species. For instance, *S. macrolepis* exhibits a blunt snout and wide mouth morphology similar to *S. barbatulum*, and lateral scale counts show overlap, with some individuals of *S. brevibarba* possessing 48–49 scales.

Cryptic diversity appears to be prevalent within several morphologically conserved *Scaphesthes* species that exhibit broad geographic distributions [4,13]. A notable example is *S. barbatulum*, which was historically regarded as widespread across southeastern mainland China and Taiwan [8]. Subsequent taxonomic revisions have recognized populations from the Jiulong River in Fujian and the middle Yangtze River basin as distinct species, *S. minnanense* and *S. brevibarba*, respectively [25,26]. Similarly, specimens collected from various river systems in Zhejiang Province, previously misidentified as *S. barbatulum*, are herein described as the new species *S. zhejiangensis*. It can be reliably distinguished from the sympatric *S. barbatulum* and other close relatives by a consistent set of morphological traits (Table 1 and Table 2) and is further supported as a distinct evolutionary lineage by molecular phylogenetic analysis (Figure 6).

The topology of the cytochrome *b* gene tree generated in this study is largely congruent with the generic-level phylogeny reconstructed previously [13], and the placement of the genus *Acrossocheilus* is consistent with prior COI-based findings [4]. However, contrary to the expectation that *S. barbatum* and *S. lini* would each be monophyletic, specimens identified as these two taxa from southern China and northern Vietnam together formed a single monophyletic clade, albeit with only moderate bootstrap support in ML (0.79) and moderate Bayesian posterior probability (0.86). Neither taxon alone was recovered as monophyletic, indicating possible conspecificity and intermixed haplotype patterns. In contrast, the samples treated herein as *S.* aff. *barbatum* from the middle Yangtze River neither clustered with *S. barbatum* and *S. lini* to form a monophyletic group, nor were they monophyletic themselves. These patterns are consistent with an unresolved taxonomic complex within this group, as reported previously [4,13]. *Scaphesthes zhejiangensis* sp. nov. was recovered as a monophyletic group and placed as the sister lineage to *S.* aff. *barbatum* from Hubei Province, with maximum bootstrap support in ML (0.98) and a Bayesian posterior probability of 1.00. The genetic distances between *S. zhejiangensis* sp. nov. and other congeneric species ranged from 3.9% to 14.0%, with a particularly notable divergence of 11.7% from the morphologically similar *S. barbatulum* (Table S2). This level of genetic differentiation provides robust molecular evidence supporting the recognition of *S. zhejiangensis* as a distinct species.

Interestingly, while *S. zhejiangensis* sp. nov. is sympatric or parapatric with *S. barbatulum*, *S. brevibarba*, and *S. minnanense* in southeastern China, it is phylogenetically more closely related to a clade comprising *S. macrolepis*, *S. virgulatum*, and the *S. barbatum* species complex. Notably, *S. virgulatum* is distributed in the Qiupu River, Anhui Province [27], a lower Yangtze tributary geographically adjacent to the known range of *S. zhejiangensis*. This phylogenetic pattern suggests that evolutionary relationships within *Scaphesthes* may not conform to a simple model of geographic proximity, indicating a more complex biogeographic history. The discovery of *S. zhejiangensis* sp. nov. provides critical data for understanding the evolutionary diversification, historical dispersal, and biogeography of this genus. Future studies utilizing genome-scale data will be essential to further resolve the phylogenetic relationships within *Scaphesthes* and the broader subfamily Acrossocheilinae, and to elucidate their evolutionary trajectories.

## 5. Comparative Material

*S. brevibarba*: SHOU202406001-011, 11 specimens, 92.0–121.5 mm SL, Huangshan City, The Yangtze River System, Anhui Province, China.

*S. barbatulum*: SHOU202407001-009, 9 specimens, 41.5–82.1 mm SL, Longquan City, Oujiang River System, Zhejiang Province, China.

*S. minnanense*: SHOU202201001-006, 6 specimens, 80.6–111.4 mm SL, Zhangzhou City, Jiulong River System, Fujian Province, China.

*S. virgulatum*: SHOU202501001-004, 4 specimens, 105.3–154.9 mm SL, Chizhou City, Qiupu River System, Anhui Province, China.

*S. barbatum*: SHOU202007001-002, 2 specimens, 84.9–94.4 mm SL, Liuzhou City, The Pearl River System, Guangxi Province, China.

*S. lini*: SHOU202212001-003, 3 specimens, 74.6–86.9 mm SL, Chaozhou City, Hanjiang River System, Guangdong Province, China; SHOU202201001-002, 2 specimens, 98.9–107.5 mm SL, Baise City, The Pearl River System, Guangxi Province, China; SHOU202210001-004, 4 specimens, 99.2–127.1 mm SL, Qiannan State, The Pearl River System, Guizhou Province, China.

*S. macrolepis*: SHOU202107001, 1 specimen, 113.8 mm SL, Nanyang City, The Yangtze River System, Henan Province, China; SHOU202208001-004, 4 specimens, 81.9–93.1 mm SL, Taian City, The Yellow River System, Shandong Province, China.

*S.* aff. *barbatum*: SHOU202412001, 1 specimen, 107.4 mm SL, Zhangjiajie City, The Yangtze River System, Hunan Province, China; SHOU202202001-002, 2 specimens, 62.1–80.3 mm SL, Qiannan State, The Pearl River System, Guizhou Province, China; SHOU202210001-002, 2 specimens, 93.6–96.7 mm SL, Liupanshui City, Wujiang River System, Guizhou Province, China.

## 6. Conclusions

This study describes *Scaphesthes zhejiangensis* as a new species of shoveljaw carp from Zhejiang Province, China. The species is distinguished from its congeners by a combination of morphological characteristics, including a longer and narrower body, a wider mouth, and specific scale counts. Molecular phylogenetic analysis based on the mitochondrial cyt *b* gene confirms its distinct taxonomic status, showing significant genetic divergence from other species, particularly from the morphologically similar *S. barbatulum*. The discovery of *S. zhejiangensis* not only enriches the biodiversity of freshwater fishes in eastern China but also highlights the importance of integrating morphological and molecular data in the identification of cryptic species within the genus *Scaphesthes*.

## Figures and Tables

**Table 1:** Morphological variation among *Scaphesthes zhejiangensis* sp. nov., *S. brevibarba*, *S. barbatulum*, and *S. minnanense*.

	*S. zhejiangensis* (n = 19)	*S. brevibarba* (n = 11)	*S. minnanense* (n = 6)	*S. barbatulum* (n = 9)
Body depth/standard length (mean ± SD) (%)	19.9–22.2 (20.8 ± 0.7)	**22.7–25.2 (23.6 ± 0.7)** ***	**23.8–24.9 (24.4 ± 0.4)** ***	**25.4–28.7 (27.5 ± 1.1)** ***
Lateral line scales (mean ± SD)	46–49 (47.4 ± 1)	45–49 (47.3 ± 1.2)	**44–45 (44.7 ± 0.5)** ***	46–47 (46.1 ± 0.3) ***
Pre-dorsal scales (mean ± SD)	15–17 (15.4 ± 0.7)	14–16 (15.1 ± 0.8)	**12–14 (13.2 ± 0.8)** ***	12–15 (13.4 ± 1) ***
Maxillary barbels	less than 1/3 of eye diameter	**Papillose**	**Longer than 1/3 of eye diameter**	less than 1/4 of eye diameter
Rostral barbels	Papilliform	**Minuscule**	**Well-developed**	Papilliform

Note: Independent-sample *t*-tests were used to compare each species with the new species. *** *p* < 0.001. Bold indicates non-overlapping measurements and counts.

**Table 2:** Morphological differences of *Scaphesthes zhejiangensis* sp. nov., *S. barbatum* complex, *S. lini* ^1^, *S. virgulatum* and *S. macrolepis*.

Characters	*S. zhejiangensis*	*S. barbatum* Complex	*S. virgulatum*	*S. macrolepis*	*S. lini*
Mouth width/head length (%)	36.2–45.3	24.8–28.5 *^2^ 29.3–38.5 ^3^	27.4–28.9 *	31.5–35.5 *	26.0–31.1 *
Head depth/head length (%)	66.8–73.3	50.4–51.7 *^2^ 55.6–64.1 *^3^	54.0–59.3 *	54.8–62.7 *	55.8–58.4 *
Last simple dorsal ray	Slender, smooth	Slender, smooth	Strong, serrated *	Slender, smooth	Strong, serrated *
Maxillary barbels	Shorter than 1/3 of eye diameter	Longer than 1/2 of eye diameter *	Longer than 1/2 of eye diameter *	Shorter than 1/3 of eye diameter	Almost equal to 1/2 of eye diameter *
longitudinal dark stripe along lateral line ^4^	Absent; blackish	Present in some individuals; grayish *	Present in some individuals; grayish *	Absent; blackish	Present in some individuals; grayish
Lateral line scales	46–49	47–49 ^5^	45–46	50–52 *	48–49 ^5^
Body depth/standard length (%)	19.9–22.2	22.2–26.3	26.3–27.1 *	22.7–27.0 *	22.7–26.3 *

* Non-overlapping measurements and counts. ^1^ According to molecular barcoding, *S. lini* and *S. barbatum* were proven to be the same species. ^2^ From *S. barbatum* collected from Guangxi (2 individuals). ^3^ From *S.* aff. *barbatum* collected from Hunan (1 individual) and Guizhou (4 individuals). ^4^ Present in *S. brevibarba*, *S. barbatulum*, and *S. minnanense*; it is also present in some individuals and is grayish in colour. ^5^ According to Shan et al. [8] due to quality of some specimens.

**Table 3:** Morphological measurements and counts for *Scaphesthes zhejiangensis* sp. nov.

Characters	Holotype	Range	Mean
Standard length (mm)	141.5	54.9–141.5	71.8
Percentages of SL (%)			
Body depth	22.2	19.9–22.2	20.8
Head length	20.6	20.6–26	24.0
Dorsal fin length	20.5	18.0–24.0	21.9
Pectoral fin length	20.2	17.4–20.3	18.6
Pelvic fin length	17.7	15.6–18.0	16.9
Anal fin length	16.0	16.0–19.0	17.2
Caudal peduncle length	17.6	17.5–20.2	19.2
Caudal peduncle depth	9.7	7.3–11.1	10.0
Predorsal length	47.6	47.4–52.2	49.5
Snout—Pectoral fins	21.8	21.8–27.0	24.6
Snout—Pelvic fins	50.0	50.0–57.3	53.2
Snout—anal fin	73.2	73.2–79.7	75.4
Percentages of HL (%)			
Head depth	66.8	66.8–73.3	70
Head width	63.9	59.5–66.6	62.1
Snout length	38.3	31.2–38.3	33.9
Mouth width	45.2	36.2–45.3	39.5
Eye diameter	24.8	24.4–29.2	27.2
Interorbital width	42.0	38.1–43.2	40.8
Meristic counts			
Dorsal fin	iii,8	iii,8	
Pectoral fins (mean ± SD; mode)	i,16	i,15–16 (15.5 ± 0.5; 16)	
Pelvic fins (mean ± SD; mode)	i,9	i,8–9 (8.8 ± 0.4; 9)	
Anal fin	iii,5	iii,5	
Lateral line scales (mean ± SD; mode)	49	46–49 (47.4 ± 1; 47)	
Scales above lateral line	7	6–7	
Scales below lateral line	4	3–4	
Predorsal scales	17	15–17 (15.4 ± 0.7; 15)	
Circumpeduncular scales	16	16	

## Data Availability

The original genetic data presented in the study are openly available in NCBI. All other data are available within the Supplementary Materials of the paper.

## Supplementary Materials

The following supporting information can be downloaded at: https://www.mdpi.com/article/10.3390/ani16081176/s1. Table S1: Sampling information of *Scaphesthes zhejiangensis* sp. nov. and comparative species; Table S2: Interspecific nucleotide divergence (K2P distances) within the genus *Scaphesthes*.
